# Pediatric Emergency Departments and Urgent Care Visits in Houston after Hurricane Harvey

**DOI:** 10.5811/westjem.2021.2.49050

**Published:** 2021-05-26

**Authors:** S. Aya Fanny, Brent D. Kaziny, Andrea T. Cruz, Elizabeth A. Camp, Kristy O. Murray, Tyler J. Nichols, Corrie E. Chumpitazi

**Affiliations:** * Baylor College of Medicine, Department of Pediatrics, Section of Emergency Medicine, Houston, Texas; † National School of Tropical Medicine, Baylor College of Medicine, Houston, Texas; ‡ Advocate Christ Medical Center, Oak Lawn, Illinois

## Abstract

**Introduction:**

Natural disasters are increasingly common and devastating. It is essential to understand children’s health needs during disasters as they are a particularly vulnerable population. The objective of this study was to evaluate pediatric disease burden after Hurricane Harvey compared to the preceding month and the same period in the previous year to inform pediatric disaster preparedness.

**Methods:**

This was a retrospective cross-sectional study of patients seen at pediatric emergency departments (ED) and urgent care centers (UCC) 30 days before (late summer) and after (early fall) the hurricane and from the same time period in 2016. We collected demographic information and the first five discharge diagnoses from a network of EDs and UCCs affiliated with a quaternary care children’s hospital in Houston, Texas. We calculated the odds of disease outcomes during various timeframes using binary logistic regression modeling.

**Results:**

There were 20,571 (median age: 3.5 years, 48.1% female) and 18,943 (median age: 3.5 years, 47.3% female) patients in 2016 and 2017, respectively. Inpatient admission rates from the ED a month after Harvey were 20.5%, compared to 25.3% in the same period in 2016 (P<0.001). In both years, asthma and other respiratory illnesses increased from late summer to early fall. After controlling for these seasonal trends, the following diseases were more commonly seen after the hurricane: toxicological emergencies (adjusted odds ratio [aOR]: 2.61, 95% [confidence interval] CI, 1.35–5.05); trauma (aOR: 1.42, 95% CI, 1.32–1.53); and dermatological complaints (aOR: 1.34, 95% CI, 1.23–1.46).

**Conclusion:**

We observed increases in rashes, trauma, and toxicological diagnoses in children after a major flood. These findings highlight the need for more medication resources and public health and education measures focused on pediatric disaster preparedness and management.

## INTRODUCTION

Floods are the most common natural disaster around the world and are becoming increasingly more frequent and devastating.^1^ Studies have reported increases in illness and healthcare needs after hurricanes and major floods. A systematic review and meta-analysis identified drowning, blunt trauma, toxic exposures, water- and vector-borne illnesses, respiratory infections, skin infections, exacerbation of chronic non-communicable illnesses, and long-lasting psychological distress after floods.^1^,^2^ After Hurricane Katrina in 2005, evacuees at a shelter in Houston, Texas, received medical care for fever, rash, gastrointestinal complaints and respiratory infections.^3^,^4^ Similar observations were reported after Hurricane Rita in Louisiana in 2005.^4^ These studies highlight the importance of improving our understanding of the clinical impact of natural disasters, particularly among vulnerable populations such as children.

Hurricane Harvey made landfall in Southeast Texas on August 25, 2017. Flooding affected about a third of the state population and was linked to 94 deaths.^5^ There are few studies that solely focus on the health effects of floods on the pediatric population.^6^ We aimed to fill this gap in the literature and describe the burden of disease in children after Hurricane Harvey in Houston, a major flooding event in one of the largest urban areas in the United States.

## MATERIALS AND METHODS

### Patients and Setting

Houston is the fourth largest city in the US, with a population of 2,325,502 as of July 2018.^7^ The Greater Houston metropolitan area covers 9,444 square miles.^7^ We conducted a retrospective cross-sectional study of patients seen at all emergency departments (ED) and urgent care centers (UCC) associated with a large, quaternary-care children’s hospital in the greater Houston area from July 26–September 23, 2016 and July 26–September 23, 2017. In 2016, the hospital network had two EDs and six UCCs and grew to three EDs and eight UCCs in 2017. We extracted demographic information (age, gender, and address), health record number, date and location of the encounter, the first five discharge diagnoses, and disposition from the electronic health record for each patient. We excluded patients who left without being seen by a provider.

### Diagnosis Code Groups

We converted *International Classification of Diseases*, 10^th^ revision (ICD-10) diagnosis codes into ICD-9 diagnosis codes using the publicly available general equivalence mappings developed by the Centers for Medicaid and Medicare Services.^8^ We then grouped ICD-9 diagnosis codes into 21 major diagnosis groups and 77 subgroups using a published diagnosis grouping system developed specifically for pediatric EDs.^9^

### Data Analysis

We compared demographic data using Pearson’s chi-squared test. For skewed continuous data, we used a non-parametric (Mann-Whitney U test) analysis. We compared the frequency of each major diagnosis group and subgroup during the following timeframes: July 26–August 24, 2016 (late summer 2016); August 25–September 23, 2016 (early fall 2016); July 26–August 24, 2017 (late summer 2017, 30 days before Harvey); and August 25–September 23, 2017 (early fall 2017, 30 days after Harvey). Since Hurricane Harvey occurred at the end of the summer, a period that coincides with seasonal changes and the back-to-school period when children tend to have more respiratory illnesses, we compared illness rates in the late summer 2016 vs early fall 2016 to obtain baseline seasonal variation in disease frequency in our population. We compared disease frequency in our population 30 days before Hurricane Harvey (late summer 2017) and 30 days after Hurricane Harvey (early fall 2017). To account for seasonal variation from year to year, we compared disease frequency in early fall 2016 vs early fall 2017. ED disposition location was analyzed for all time periods.

**Population Health Research Capsule**

What do we already know about this issue?

*Natural disasters are increasingly common. Studies report increases in illness and healthcare needs in the aftermath, yet few have focused on pediatric health.*

What was the research question?

*Our goal was to evaluate pediatric visits after Hurricane Harvey in Houston, Texas, to inform disaster preparedness.*

What was the major finding of the study?

*We found increases in rashes, trauma, and toxicological diagnoses in children after a major flooding event.*

How does this improve population health?

*These findings highlight the need for more medication resources, as well as public health and education measures focused on pediatric disaster preparedness.*

We compared overall patient demographics and clinical factors to all study timeframes using Pearson’s chi-squared tests and Kruskal-Wallis test for skewed continuous data (age). Any co-factor with a *P*-value < 0.20 (age, gender, race, ethnicity, insurance status, and encounter location) was considered for further adjustment in all subsequent models for comparative purposes. We also analyzed binary comparisons of timeframe groups between study factors. Comparisons between disease rates and timeframes were calculated using unadjusted odds ratios (OR) to provide an effect estimate. Any unadjusted OR with a *P*-value < 0.05 was further adjusted using binary logistic regression and any co-factor with a *P*-value < 0.20. We defined statistical significance as *P*-value < 0.05. Statistical analysis was conducted using the Statistical Package for the Social Sciences (SPSS), version 24 (IBM Corp., Armonk, NY). The study protocol was exempted from informed consent and approved by our local institutional review board.

## RESULTS

### Baseline Seasonal Differences: Late Summer 2016 vs Early Fall 2016

There were more patients seen in the early fall than in the late summer: 11,995 vs 8,576 (n = 20,571 total patient visits). A total of 38,860 individual diagnoses were included in these analyses: 16,072 in late summer and 22,788 in early fall. ED inpatient admission and discharge rates were comparable in the late summer and early fall (Table). In the fall, more children were seen at UCCs (57.1% vs 52.2%) and were older by one median year (3.0 vs 4.0). The following diagnosis groups were more common in early fall as compared to late summer: respiratory diseases ([adjusted odds ratio] aOR: 1.53, 95% [confidence interval] CI, 1.42–1.66); ear nose and throat (ENT)/dental/mouth diseases (aOR: 1.30, 95% CI, 1.22–1.38); neurologic diseases (aOR: 1.14, 95% CI, 1.04–1.25); asthma (aOR: 1.81, 95% CI, 1.52–2.14); infectious respiratory diseases (aOR: 1.70, 95% CI, 1.46–1.99); infectious nose and sinus disorders/upper respiratory infection (URI) (aOR: 1.58, 95% CI, 1.41–1.76); and other respiratory diseases (aOR: 1.34, 95% CI, 1.20–1.50) after adjusting for age, ethnicity, insurance status, and location (Figure, Appendix A, and Supplement)

### Seasonal Differences Year of Hurricane: Late Summer 2017 vs Early Fall 2017

There were more patients seen in the late summer of 2017 (30 days pre-hurricane) than in the early fall (30 days post-hurricane) of 2017: 9,843 (52%) vs 9,100 (48%) (n = 18,943 total patient visits). A total of 34,609 individual diagnoses were included in these analyses: 17,957 (51.9%) in late summer and 16,652 (48.1%) in early fall. Although age was statistically significant, with patients seen in early fall older by one median year, there were no significant clinically relevant differences between demographic factors and 30-day time intervals (Table). ED inpatient admission and discharge rates were comparable in the late summer and early fall. The following diagnoses were more common after Hurricane Harvey: respiratory diseases (aOR: 1.32, 95% CI, 1.22–1.44); musculoskeletal and connective tissue diseases (aOR: 1.17, 95% CI, 1.03–1.34); ENT/dental/mouth diseases (aOR: 1.16, 95% CI, 1.10–1.23); asthma (aOR: 1.81, 95% CI, 1.54–2.14), bronchospasm/wheezing (aOR: 1.73, 95% CI, 1.24–2.42); infectious nose and sinus disorders/URI (aOR: 1.53, 95% CI, 1.38–1.69); and infectious respiratory diseases (aOR: 1.22, 95% CI, 1.04–1.43) after adjusting for age, ethnicity, insurance status, and location (Figure, Appendix B, and Supplement)

### Differences Between Year of Hurricane and Prior Year: Early Fall 2016 vs Early Fall 2017

There were 21,095 patients seen: 11,995 (56.9%) in the early fall 2016 and 9,100 (43.1%) in the early fall 2017. A total of 39,440 diagnoses were included in these analyses: 22,788 (57.8%) in early fall 2016 and 16,652 (42.2%) in early fall 2017. While the absolute number of patient encounters in 2017 decreased compared to 2016, a larger proportion of those encounters took place at UCCs in 2017 than in 2016 (60.8% vs 57.1%). In the month after Harvey, a higher proportion of ED patients were discharged home than in the same period the prior yea. Conversely, the proportion of ED patients requiring surgical intervention in the month after Harvey doubled compared to the same period in 2016 (Table). The following diagnosis groups were more common after Hurricane Harvey in 2017 than in the same timeframe in 2016: toxicological emergencies (aOR: 2.61, 95% CI, 1.35–5.05); trauma (aOR: 1.42, 95% CI, 1.32–1.53); skin, dermatologic and soft tissue diseases (aOR: 1.34, 95% CI, 1.23–1.46); lacerations and amputations (aOR: 1.78, 95% CI, 1.50–2.11); contusions and abrasions (aOR: 1.93, 95% CI, 1.59–2.35); and infectious skin and dermatologic and soft tissue diseases (aOR: 1.34, 95% CI, 1.19–1.51) after adjusting for age, ethnicity, insurance status, and location (Figure, Appendix C, and Supplement)

## DISCUSSION

In this study we compared data from the 30-day period immediately following Hurricane Harvey to baseline data from the month and the year before the hurricane. Similar to other studies, we saw an increase in URIs, asthma exacerbations, trauma, toxicological emergencies, and skin rashes in the 30 days that followed the hurricane.^1^–^3^,^6^,^10^–^12^ Unlike other studies, we did not detect an increase in water- and vector-borne illnesses and gastroenteritis.^1^–^3^, ^10^,^11^ As expected, we saw an increase in URIs and asthma exacerbations in the early fall 2016, which coincides with the back-to-school period. In early fall 2016 and 2017, there was a similar increase in the frequency of asthma exacerbations and URIs. The increased odds of visiting the ED or UCC for URI or asthma during the 30-day period after Hurricane Harvey may have been due to exposure to contaminated flood waters and surfaces. Furthermore, this trend may have been compounded by the closure of primary care offices. Another plausible explanation for the nearly fourfold increase in the odds of children presenting to our EDs and UCCs with asthma after Hurricane Harvey may have been that patients were displaced without their asthma medications.

While other studies report increased indoor airborne mold levels and respiratory illnesses after floods, we were unable to specifically evaluate the impact of mold in the context of the current study due to the nature of diagnosis codes.^13^–^16^ Due to the flooding of approximately 200,000 homes and apartment buildings during Hurricane Harvey in the Greater Houston area, mold exposure was likely increased in our population.^17^ Although the Institute of Medicine reported an association between exposure to damp environments and high indoor mold levels and respiratory symptoms in certain populations such as individuals with asthma or allergies, there is no definitive scientific evidence of a causal relationship between mold exposure and respiratory illnesses to date.^18^

The marked increase in odds of toxicological emergencies (over double) and trauma (almost double the rate of lacerations) in the early fall 2017 compared to early fall 2016 reflects heightened threats to children’s safety and health during major disasters. Toxicological emergencies and trauma after floods have been reported in previous studies in the general population.^2^,^15^ However, there are special considerations for the pediatric population. Families with children should be encouraged to seek safe and early evacuation means when advised and feasible. Additionally, there is a crucial need for childproofed temporary accommodations, adult supervision and appropriate childcare before, during, and after major disasters as caretakers are busy and preoccupied with securing food, seeking shelter, and repairing damages.

Multiple factors may have contributed to the decrease in the number of visits after Harvey despite an increased number of sites. The National Hurricane Center reported that about 40,000 individuals were relocated to shelters across Texas or Louisiana, which means that many of the children who usually access care at our facilities might have been temporarily displaced.^19^ Additionally, many major highways and up to half a million cars were flooded during Harvey, which would have prevented families that did not evacuate from visiting our EDs and UCCs.^19^

Those patients may have sought care in more accessible facilities such as smaller, freestanding EDs or UCCs, or clinics that were operating within or near shelters.^11^,^20^,^21^ This possible explanation is supported by a study from the closest major metropolitan area that reported an 11% increase in ED visits in the Dallas-Fort Worth area in the month following Hurricane Harvey, with an associated increase in patients who reported residing in the metro Houston area and presented with chief complaints that included the words “hurricane,” “Harvey,” “evacuee,” or “evacuate.”^22^ A similar decrease in total ED patient visits at a large county hospital in the metro Houston area in the four weeks following Hurricane Harvey was noted.^23^

Conversely, the decrease in percentage of inpatient admissions in early fall 2017 compared to the same period of the prior year may reflect the fact that in the immediate period following Hurricane Harvey, many primary care offices were closed and families that had access to transportation used our EDs and UCCs for non-urgent care. Unlike other medical facilities, our facilities did not incur any physical damage, flooding, or loss of power during the storm. Our hospital system activated ride-out teams that arrived at our various hospitals before the storm; so, we had appropriate staffing and maintained our usual bed capacity for inpatient care.

## LIMITATIONS

Our study period extends only 30 days after Hurricane Harvey, so our results are limited and do not reflect the long-term health effects of this major catastrophic event on the pediatric population. A few studies highlight the short- and long-term mental health effects of Hurricane Harvey, and this remains an important and active research area.^11^,^20^,^24^,^25^ With such a high rate of toxicological emergencies and physical trauma in our study population, it is highly likely that many of those patients would also be at risk for anxiety, post-traumatic stress disorder, depression, and other mental health conditions that may not have been diagnosed in the ED or UCC setting.^11^,^20^,^24^,^26^,^27^ Furthermore, although this study includes over 38,000 patient encounters, it is limited to one hospital network and does not include patients who may have sought care in other facilities or may not have had access to care. While this is a limitation, it is important to note that our hospital network provides care for nearly two-thirds of the pediatric population in our metropolitan area.

## CONCLUSION

Hurricane Harvey was the second most devastating flood in US history after Hurricane Katrina in 2005, affecting hundreds of thousands of families. Such major disasters are becoming increasingly common in the US and around the world. This study specifically reports on some of the health issues children face in the immediate period after a major flood. Our results highlight the urgent need for more resources devoted to pediatric disaster-preparedness efforts. Such efforts should include increased public health campaigns focused on injury prevention and flood water exposure avoidance aimed at families with children, stockpiling of pediatric trauma care equipment and medications, reinforced capability of emergency medical services and EDs to provide care to pediatric trauma patients and recognize toxicological emergencies in pediatric patients. More research is needed on the long-term effects of major floods on children to inform disaster prevention and management strategies and policies.

## Supplementary Information









## Figures and Tables

**Figure:**
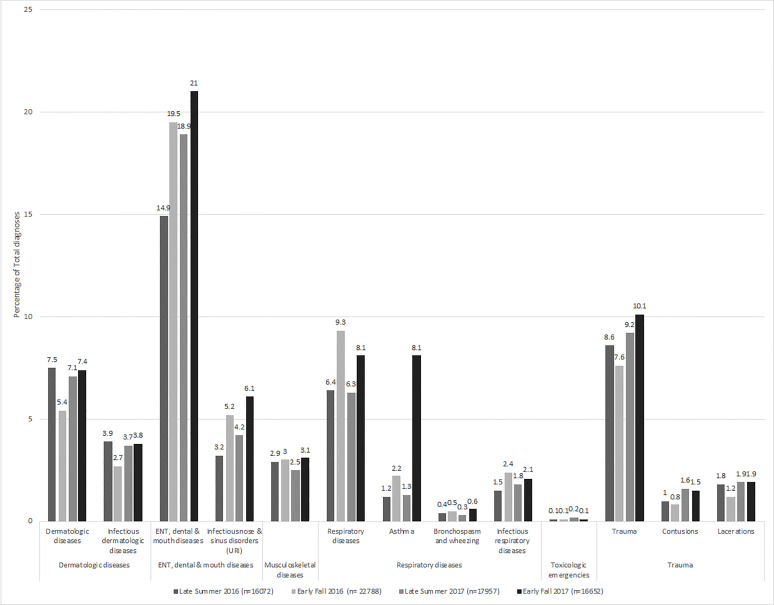
Emergency department diagnosis groups by year, before and after Hurricane Harvey which made landfall on August 25, 2017.

**Table:** Patient demographics in 2016 and 2017, before and after Hurricane Harvey which made landfall on August 25, 2017 (n = 39,514 patient visits).

	Late summer 2016 N = 8,576 (21.7%) N (%) or median (IQR)	Early fall 2016 N = 11,995 (30.4%) N (%) or median (IQR)	Late summer 2017 N = 9,843 (24.9%) N (%) or median (IQR)	Early fall 2017 N = 9,100 (23.0%) N (%) or median (IQR)	P-value^a^
Age	3.0 (1.33, 9.0)	4.0 (1.56, 10.0)	3.0 (1.50, 9.0)	4.0 (1.55, 9.0)	<0.001
Gender					0.23
Female	4,169 (48.6)	5,725 (47.7)	4,648 (47.2)	4,306 (47.3)	
Male	4,407 (51.4)	6,270 (52.3)	5,195 (52.8)	4,794 (52.7)	
Race					0.84
White	6,154 (77.2)	8,558 (77.0)	7,035 (77.1)	6454 (76.3)	
Black	1,389 (17.4)	1,914 (17.2)	1,581 (17.3)	1,528 (18.1)	
Asian	399 (5.0)	592 (5.3)	477 (5.2)	436 (5.2)	
Other^b^	29 (0.4)	51 (0.5)	35 (0.4)	36 (0.4)	
Ethnicity					0.10
Non-Hispanic	4,486 (55.2)	6,214 (54.6)	5,141 (55.0)	4,623 (53.5)	
Hispanic	3,640 (44.8)	5,162 (45.4)	4,198 (45.0)	4,020 (46.5)	
Insurance Status					<0.001
Public	3,740 (43.6)	5,107 (42.6)	3,975 (40.4)	3,867 (42.5)	
Private	3,317 (38.7)	4,696 (39.1)	3,555 (36.1)	3,061 (33.6)	
Self-pay	1,500 (17.5)	2,170 (18.1)	2,268 (23.0)	2,151 (23.6)	
International/other	19 (0.2)	22 (0.2)	45 (0.5)	21 (0.2)	
Location					<0.001
ED	4,100 (47.8)	5,145 (42.9)	3,676 (37.3)	3,563 (39.2)	
UCC	4,476 (52.2)	6,850 (57.1)	6,167 (62.7)	5,537 (60.8)	
ED disposition					<0.001
Discharge	2,977 (72.6)	3,764 (73.2)	2,820 (76.7)	2,768 (77.7)	
Admit	1,082 (26.4)	1,302 (25.3)	799 (21.7)	730 (20.5)	
Surgery	13 (0.3)	17 (0.3)	27 (0.7)	24 (0.7)	
Other^c^	28 (0.7)	62 (1.2)	30 (0.8)	41 (1.2)	

a P-values were calculated using Pearson’s chi-squared test or the Kruskal-Wallis test.

b Other includes American Indian, Alaskan Native, Native Hawaiian, and other Pacific Islander.

c Other includes transfers, left against medical advice, left without being seen and death.

*IQR*, interquartile range; *ED*, emergency department; *UCC*, urgent care center.
