# Supplementary material for: Pediatric Emergency Departments and Urgent Care Visits in Houston after Hurricane Harvey
Source: West J Emerg Med. 2021 May 26;22(3):763–8. doi: 10.5811/westjem.2021.2.49050 (PMC8203006; doi:10.5811/westjem.2021.2.49050)
Supplement: Supplementary file 1 [file wjem-22-763-s001.docx]

**Appendix A**

**Table A.** Select major diagnosis groups and subgroups frequency in late summer vs early fall 2016 (N = 38,860 diagnoses).

|  | **Late Summer 2016**  **N = 16072**  **n (%)** | **Early Fall 2016**  **N = 22788**  **n (%)** | **OR** | **aOR^a^** | **95% CI** | ***P*-value** |
| --- | --- | --- | --- | --- | --- | --- |
| Respiratory diseases | 1034 (6.4) | 2116 (9.3) | 1.49 | 1.53 | 1.42 – 1.66 | <0.001 |
| Asthma | 193 (1.2) | 491 (2.2) | 1.81 | 1.81 | 1.52 – 2.14 | <0.001 |
| Infectious respiratory diseases | 239 (1.5) | 552 (2.4) | 1.65 | 1.70 | 1.46 – 1.99 | <0.001 |
| Other respiratory diseases | 529 (3.3) | 957 (4.2) | 1.29 | 1.34 | 1.20 – 1.50 | <0.001 |
| ENT/dental/mouth diseases | 2387 (14.9) | 4438 (19.5) | 1.39 | 1.30 | 1.22 – 1.38 | <0.001 |
| Infectious nose and sinus diseases/URI | 522 (3.2) | 1178 (5.2) | 1.62 | 1.58 | 1.41 – 1.76 | <0.001 |
| Neurological diseases | 876 (5.5) | 1411 (6.2) | 1.15 | 1.14 | 1.04 – 1.25 | 0.004 |

^a^Odds ratios were adjusted for age, ethnicity, insurance status, and location.

*aOR*, adjusted odds ratio; *CI*, confidence interval; *ENT*, ear, nose, and throat; *OR*, odds ratio; *URI*, upper respiratory infection.
