# Supplementary material for: Pediatric Emergency Departments and Urgent Care Visits in Houston after Hurricane Harvey
Source: West J Emerg Med. 2021 May 26;22(3):763–8. doi: 10.5811/westjem.2021.2.49050 (PMC8203006; doi:10.5811/westjem.2021.2.49050)
Supplement: Supplementary file 2 [file wjem-22-763-s002.docx]

**Appendix B**

**Table B.** Select major diagnosis groups and subgroups frequency in late summer vs early fall 2017 (N = 34,609 diagnoses).

|  | **Late Summer 2017**  **N = 17,957**  **n (%)** | **Early Fall 2017**  **N = 16,652**  **n (%)** | **OR** | **aOR^a^** | **95% CI** | ***P*-value** |
| --- | --- | --- | --- | --- | --- | --- |
| Respiratory diseases | 1132 (6.3) | 1347 (8.1) | 1.31 | 1.32 | 1.22 – 1.44 | <0.001 |
| Asthma | 237 (1.3) | 391 (2.3) | 1.80 | 1.81 | 1.54 – 2.14 | <0.001 |
| Bronchospasm and wheezing | 60 (0.3) | 93 (0.6) | 1.68 | 1.73 | 1.24 – 2.42 | 0.001 |
| Infectious respiratory diseases | 316 (1.8) | 348 (2.1) | 1.19 | 1.22 | 1.04 – 1.43 | 0.02 |
| Musculoskeletal and connective tissue diseases | 456 (2.5) | 508 (3.1) | 1.21 | 1.17 | 1.03 – 1.34 | 0.02 |
| ENT/dental/mouth diseases | 3394 (18.9) | 3496 (21.0) | 1.14 | 1.16 | 1.10 – 1.23 | <0.001 |
| Infectious nose and sinus diseases/URI | 759 (4.2) | 1013 (6.1) | 1.47 | 1.53 | 1.38 – 1.69 | <0.001 |
| Trauma | 1660 (9.2) | 1687 (10.1) | 1.11 | 1.10 | 1.02 – 1.18 | 0.01 |

^a^Odds ratios were adjusted for age, ethnicity, insurance status, and location.

*aOR*, adjusted odds ratio; *CI*, confidence interval; *ENT*, ear, nose, and throat; *OR*, odds ratio; *URI*, upper respiratory infection.
