# Supplementary material for: Pediatric Emergency Departments and Urgent Care Visits in Houston after Hurricane Harvey
Source: West J Emerg Med. 2021 May 26;22(3):763–8. doi: 10.5811/westjem.2021.2.49050 (PMC8203006; doi:10.5811/westjem.2021.2.49050)
Supplement: Supplementary file 3 [file wjem-22-763-s003.docx]

**Appendix C**

**Table C.** Select major diagnosis groups and subgroups frequency in early fall 2016 vs early fall 2017 (N = 39,440 diagnoses).

|  | **Early Fall 2016**  **N = 22,788**  **n (%)** | **Early Fall 2017**  **N = 16,652**  **n (%)** | **OR** | **aOR^a^** | **95% CI** | **p-value** |
| --- | --- | --- | --- | --- | --- | --- |
| Toxicological emergencies | 15 (0.1) | 24 (0.1) | 2.19 | 2.61 | 1.35 – 5.05 | 0.004 |
| Trauma | 1732 (7.6) | 1687 (10.1) | 1.37 | 1.42 | 1.32 – 1.53 | <0.001 |
| Lacerations and amputations | 266 (1.2) | 321 (1.9) | 1.66 | 1.78 | 1.50 – 2.11 | <0.001 |
| Contusions and abrasions | 188 (0.8) | 255 (1.5) | 1.87 | 1.93 | 1.59 – 2.35 | <0.001 |
| Skin, dermatologic, and soft tissue diseases | 1230 (5.4) | 1228 (7.4) | 1.40 | 1.34 | 1.23 – 1.46 | <0.001 |
| Infectious skin, dermatologic, and soft tissue diseases | 615 (2.7) | 629 (3.8) | 1.42 | 1.34 | 1.19 – 1.51 | <0.001 |
| Others (including screening exams, labs, devices and complications, etc) | 377 (1.7) | 337 (2.0) | 1.23 | 1.29 | 1.11 – 1.51 | 0.001 |

^a^Odds ratios were adjusted for age, ethnicity, insurance status, and location.

*aOR*, adjusted odds ratio; *CI*, confidence interval; *OR*: odds ratio.
