# Supplementary material for: Pediatric Emergency Departments and Urgent Care Visits in Houston after Hurricane Harvey
Source: West J Emerg Med. 2021 May 26;22(3):763–8. doi: 10.5811/westjem.2021.2.49050 (PMC8203006; doi:10.5811/westjem.2021.2.49050)
Supplement: Supplementary file 4 [file wjem-22-763-s004.docx]

**Supplementary Table 1.** Supplementary of adjusted regression by diagnoses (N = 33,741), with referent group the period immediately after the hurricane (Fall 2017).

| Diagnosis | Time Period | aOR^a^ | 95% CI | P-value |
| --- | --- | --- | --- | --- |
| **ENT, Dental, Mouth** | Late Summer 2016  Early Fall 2016  Late Summer 2017  Early Fall 2017 | 0.79  1.01  0.89  Ref | 0.68 – 0.91  0.89 – 1.15  0.77 – 1.02  -- | 0.001  0.92  0.10  -- |
| Nose & Sinus Disorders | Late Summer 2016  Early Fall 2016  Late Summer 2017  Early Fall 2017 | 0.63  1.07  0.65  Ref | 0.50 – 0.79  0.89 – 1.29  0.51 – 0.82  -- | <0.001  0.47  <0.001  -- |
| **Endocrine, Metabolic** | Late Summer 2016  Early Fall 2016  Late Summer 2017  Early Fall 2017 | 1.29  1.09  1.15  Ref | 1.05 – 1.58  0.89 – 1.33  0.92 – 1.43  -- | 0.02  0.42  0.21  -- |
| **GI Diseases** | Late Summer 2016  Early Fall 2016  Late Summer 2017  Early Fall 2017 | 1.13  1.14  1.00  Ref | 1.03 – 1.24  1.05 – 1.25  0.91 – 1.11  -- | 0.01  0.003  0.93  -- |
| Abdominal Pain | Late Summer 2016  Early Fall 2016  Late Summer 2017  Early Fall 2017 | 1.41  1.47  0.96  Ref | 1.14 – 1.73  1.21 – 1.78  0.77 – 1.20  -- | 0.001  <0.001  0.72  -- |
| **Genital & Reproductive Diseases** | Late Summer 2016  Early Fall 2016  Late Summer 2017  Early Fall 2017 | 1.34  1.05  0.90  Ref | 1.03 – 1.75  0.81 – 1.37  0.67 – 1.21  -- | 0.03  0.72  0.48  -- |
| **Musculoskeletal & Connective Diseases** | Late Summer 2016  Early Fall 2016  Late Summer 2017  Early Fall 2017 | 1.33  1.19  0.84  Ref | 1.10 – 1.61  0.99 – 1.43  0.68 – 1.03  -- | 0.003  0.06  0.10  -- |
| **Neurological Diseases** | Late Summer 2016  Early Fall 2016  Late Summer 2017  Early Fall 2017 | 1.35  1.38  1.29  Ref | 1.17 – 1.55  1.20 – 1.58  1.11 – 1.49  -- | <0.001  <0.001  0.001  -- |
| **Respiratory Diseases** | Late Summer 2016  Early Fall 2016  Late Summer 2017  Early Fall 2017 | 0.80  1.34  0.68  Ref | 0.71 – 0.90  1.20 – 1.49  0.59 – 0.78  -- | <0.001  <0.001  <0.001  -- |
| Asthma | Late Summer 2016  Early Fall 2016  Late Summer 2017  Early Fall 2017 | 0.38  0.82  0.50  Ref | 0.30 – 0.48  0.68 – 0.98  0.40 – 0.63  -- | <0.001  0.03  <0.001  -- |
| Bronchospasm & Wheezing | Late Summer 2016  Early Fall 2016  Late Summer 2017  Early Fall 2017 | 0.52  0.86  0.56  Ref | 0.29 – 0.92  0.54 – 1.37  0.31 – 1.01  -- | 0.02  0.52  0.05  -- |
| Infectious Respiratory Diseases | Late Summer 2016  Early Fall 2016  Late Summer 2017  Early Fall 2017 | 0.82  1.46  0.69  Ref | 0.64 – 1.06  1.17 – 1.82  0.53 – 0.92  -- | 0.13  0.001  0.01  -- |
| Other Respiratory Diseases | Late Summer 2016  Early Fall 2016  Late Summer 2017  Early Fall 2017 | 1.33  1.89  0.95  Ref | 1.10 – 1.60  1.59 – 2.24  0.77 – 1.17  -- | 0.003  <0.001  0.63  -- |
| **Skin, Dermatologic & Soft Tissue Diseases** | Late Summer 2016  Early Fall 2016  Late Summer 2017  Early Fall 2017 | 0.97  0.76  0.92  Ref | 0.85 – 1.11  0.66 – 0.87  0.80 – 1.06  -- | 0.66  <0.001  0.24  -- |
| Infectious Skin, Dermatologic & Soft Tissue | Late Summer 2016  Early Fall 2016  Late Summer 2017  Early Fall 2017 | 1.05  0.82  0.86  Ref | 0.87 – 1.27  0.67 – 0.99  0.70 – 1.06  -- | 0.62  0.04  0.17  -- |
| **Systemic States*** | Late Summer 2016  Early Fall 2016  Late Summer 2017  Early Fall 2017 | 1.33  1.49  1.25  Ref | 1.09 – 1.63  1.23 – 1.80  1.01 – 1.55  -- | 0.01  <0.001  0.04  -- |
| **Toxicologic Emergencies** | Late Summer 2016  Early Fall 2016  Late Summer 2017  Early Fall 2017 | 0.29  0.31  1.49  Ref | 0.13 – 0.66  0.15 – 0.64  0.88 – 2.52  -- | 0.003  0.001  0.14  -- |
| **Trauma** | Late Summer 2016  Early Fall 2016  Late Summer 2017  Early Fall 2017 | 0.91  0.79  0.97  Ref | 0.81 – 1.01  0.71 – 0.88  0.87 – 1.08  -- | 0.07  <0.001  0.57  -- |
| Lacerations & Amputations | Late Summer 2016  Early Fall 2016  Late Summer 2017  Early Fall 2017 | 0.92  0.64  1.12  Ref | 0.73 – 1.17  0.51 – 0.82  0.90 – 1.41  -- | 0.51  <0.001  0.32  -- |
| Contusions & Abrasions | Late Summer 2016  Early Fall 2016  Late Summer 2017  Early Fall 2017 | 0.56  0.38  1.09  Ref | 0.43 – 0.73  0.29 – 0.50  0.87 – 1.37  -- | <0.001  <0.001  0.47  -- |
| Complications of Trauma | Late Summer 2016  Early Fall 2016  Late Summer 2017  Early Fall 2017 | 0.20  0.06  0.50  Ref | 0.06 – 0.74  0.01 – 0.43  0.18 – 1.34  -- | 0.02  0.01  0.17  -- |

^a^Adjusted for age, gender, location and emergency department disposition.

Late summer 2016: July 26^th^- August 24^th^ 2016, Early fall 2016: August 25^th^- September 23^rd^ 2016, Late summer 2017 (30 days before Harvey): July 26^th^- August 24^th^ 2017, and Early fall 2017 (30 days after Harvey): August 25^th^- September 23^rd^ 2017.
